# The prognostic significance of combined androgen receptor, E-Cadherin, Ki67 and CK5/6 expression in patients with triple negative breast cancer

**DOI:** 10.18632/oncotarget.20293

**Published:** 2017-08-16

**Authors:** Barbara Adamo, Giuseppina Rosaria Rita Ricciardi, Antonio Ieni, Tindara Franchina, Carmine Fazzari, Maria Vita Sanò, Giuseppe Angelico, Caruso Michele, Giovanni Tuccari, Vincenzo Adamo

**Affiliations:** ^1^ Department of Medical Oncology, Hospital Clínic of Barcelona, Barcelona, Spain; ^2^ Medical Oncology Unit A.O. Papardo & Department of Human Pathology University of Messina, Messina, Italy; ^3^ Department of Human Pathology of Adult and Evolutive Age “Gaetano Barresi”, Section of Pathology, University of Messina, AOU Policlinico ”G. Martino“ Messina, Italy; ^4^ Pathology Unit, Humanitas Center of Oncology, Catania, Italy; ^5^ Medical Oncology, Humanitas Catania Oncology Center, Catania, Italy; ^6^ G. F. Ingrassia Department, Section of Anatomic Pathology, University Hospital “Policlinico-Vittorio Emanuele”, Catania, Italy

**Keywords:** androgen receptor, triple negative breast cancer, e-cadherin, Ki67, CK5/6

## Abstract

**Background:**

Triple Negative Breast Cancer (TNBC) represents a heterogeneous group of tumors with poor prognosis owing to aggressive tumor biology and lack of targeted therapies. No clear prognostic biomarkers have been identified to date for this subgroup.

**Materials and Methods:**

In this retrospective study we evaluated the prognostic role of 4 different molecular determinants, including androgen receptor (AR), E-cadherin (CDH1), Ki67 index, and basal cytokeratins (CKs) 5/6, in a cohort of 99 patients with TNBC. All patients received neo/adjuvant chemotherapy (mostly anthracycline/taxane-based). Immunohistochemistry (IHC) was performed in formalin-fixed paraffin-embedded primary tumor samples. CDH1 expression was considered positive as ≥ 30% of the membrane cells staining. AR positivity was defined as > 10% of positive tumor cells. High Ki67 was defined as ≥20% positive tumor cells. CK5/6 expression was judged positive if the score was ≥1.

**Results:**

The absence of AR expression was significantly associated with highly undifferentiated tumors. Univariate analyses showed that lack of expression of CDH1, tumor size and nodal status were significantly correlated with worse RFS and OS (p< 0.05). AR expression and low Ki67 showed a trend towards better RFS and OS. Patients with absent CK5/6 expression in univariate and multivariate analyses had poorer RFS (p=0.02 and p=0.002, respectively) and OS (p=0.05 and p=0.02, respectively). Multivariate analysis showed an independent association between CDH1 expression and better RFS and OS (p< 0.05) beyond tumor size, nodal status, and grade. The Kaplan-Meier curves showed that patients with AR and CDH1 negative expression and high Ki-67 levels have a significant correlation with poor outcome.

**Conclusions:**

Our study supports the use of IHC expression of AR, CDH1, Ki67, and CK5/6 as prognostic markers in TNBCs and suggests a link between their expression and prognosis and may help to stratify TNBC patients in different prognostic classes.

## INTRODUCTION

Triple negative breast cancer (TNBC) represents 10-15% of all breast cancer (BC) subtypes and is defined by the lack of immunohistochemical expression of estrogen receptor, progesterone receptor and absence of overexpression and/or amplification of c-ErbB2 by immunohistochemistry (IHC) or fluorescence *in situ* hybridization (FISH) [1–3]

TNBC is a highly aggressive disease with a poorer prognosis compared to other subtypes of BC and draws no benefits from endocrine and anti-HER2 therapies [4] characterized by shorter disease free intervals and overall survival in the metastatic setting [5].

These tumors occur typically in young pre-menopausal African-American women and are identified as highly undifferentiated tumors with a high proliferation index and early, more frequent visceral or central nervous system metastases relapse than other subtypes [6–8].

Although most TNBCs have a ductal histology, other tumor histological types may occur, including metaplastic [9], medullary [10], adenoic cystic [11], apocrine [12], and secretory carcinomas [13].

TNBC is an inter and intra-tumor heterogeneous disease that presents distinct biomolecular prognostic and therapeutic features [14, 15].

The TNBC population frequently presents BRCA1/2, TP53(62%) and PI3KCA mutations (10.2%) [16–18].

The development of gene expression signatures has allowed a better understanding of the heterogeneity of TNBC with different classification systems [19, 20].

Recently, Lehmann et al. identified at least 6 different molecular subtypes of TNBC through gene expression profiles (GEP) of 21 data sets of breast cancer, including two basal-like (BL1 and BL2), an immunomodulatory (IM), a mesenchymal (M), a mesenchymal stem cell-like (MSL), and a luminal androgen receptor (LAR) [21]. The expression of specific genes and pathways characterizes the different molecular subtypes: elevated expression of genes involved in the cell cycle and DNA damage response are enriched in the BL1 subtype; the BL2 subtype is associated with growth factor signaling and myoepithelial markers; the M and MSL subtypes are enriched with genes involved in epithelial-to-mesenchymal transition (EMT) and growth factor pathways, although the MSL subtype has decreased expression of genes involved in proliferation.

The IM subtype is defined by the expression of immune antigens and genes involved in cytokine and core immune signal transduction pathways; and, finally, the LAR subtype is characterized by luminal gene expression and androgen receptor (AR) pathway [22].

These molecular subtypes have different clinical outcomes. Indeed, the relapse-free survival (RFS) is significantly lower in the LAR subtype with no difference in terms of distant-metastasis-free survival (DMFS) between these subtypes. Although the LAR subtype is characterized by a shorter RFS, the failure to increase the DMFS suggests that this subtype has a greater propensity for locoregional recurrence [21].

The different prognostic behavior of these subtypes has been recently confirmed by the gene expression analysis of the Cancer Genome Atlas (TCGA). This study showed that median OS and DFS of patients in the group BL1, IM and MSL were almost double than those of patients with tumors BL2, LAR and M subtypes [16].

More recently, Ring et al. based on minimal gene sets to clinically subtype TNBC patients, refined the molecular classification of triple negative tumors. This novel expression algorithm, reduced to 101 genes (versus the original 2188-gene expression algorithm), reproduced the original classification of Lehmann et al. and was highly concordant in both the same set of seven TNBC cohorts used to generate the TNBC type algorithm (87 %), and in an independent cohort (88 %) [23].

Another recent genomic analysis using DNA and RNA profiling of 198 TNBC tumors has identified four subtypes with distinct prognosis: basal like immune-activated (BLIA), basal-like immunosuppressed (BLIS), mesenchymal (MES) and luminal androgen receptor (LAR) subgroups. The best and the worst clinical outcomes for RFS and OS have been observed in patients with BLIA and BLIS, respectively. In addition, Burstein et al. identified, for each specific molecular subtype, new biomarkers and potential therapeutic targets: the androgen receptor, MUC1 and several genes regulated by estrogen for the LAR subgroup; IGF1, prostaglandin F receptor for the MES subtype; SOX transcription factors and immunoregulatory molecule VTCN1 for the BLIS subtype; and STAT trascription factor for the BLIA subtypes [24].

These data provides the rationale for the study of new and potential therapeutic targets pharmacologically exploitable [22]. Today, cytotoxic chemotherapy remains the mainstay of treatment for this disease lacking of FDA approved targeted therapies.

There is an urgent unmet medical need to identify and develop new efficient prognostic markers that may predict the outcome of TNBC patients and may allow to further stratifying this tumor subtype in more homogeneous subgroups.

The future management and treatment decision making in TNBC will set on individualized tailored treatments based on the identification of prognostic and predictive biomarkers ably to predict patients’ outcome. In the last few years, several studies have attempted to identify novel potential prognostic biomarkers, with controversial results.

The aim of our study is to evaluate the potential prognostic role of Androgen receptor (AR), E-Cadherin (CDH1), Ki-67, and CK5/6 and to investigate the correlation between these molecular determinants expression and the clinical outcome.

## RESULTS

In total, TNBC samples taken from 99 patients were included in the present study and analyzed for androgen receptor, E-cadherin, Ki 67 and CK5/6 expression. Patient and tumor characteristics are listed in Table 1. Median age was 61 years (range 33-83).

**Table 1:** Baseline characteristics of our cohort of patients

Variable	N(%)
Total	99
Median age	61 years (range 33-83)
Surgery	
Mastectomy	34 (34.3%)
Tumorectomy/quadrantectomy	65 (65.7%)
Lymph-nodal sentinel biopsy	21 (21.2%)
Dissection of axillary lymph nodes	78 (78.8%)
Hystological Type	
Ductal	85 (85.8%)
Lobular	10 (10.1%)
Medullary	4 (4.1%)
Grade	
G2	33 (33.3%)
G3	66 (66.7%)
Nodal status	
N0	49 (49.5%)
N+	50 (50.5%)
Tumor stage	
I	32 (32.3%)
II	37 (37.4%)
III	30 (30.3%)

Of 99 patients, 34 are underwent mastectomy and 65 tumorectomy/quadrantectomy. The lymph-nodal sentinel biopsy was performed in 21.2% of cases, whereas the dissection of axillary lymph nodes was performed in 78.8% patients.

The majority of tumors was ductal (n = 85, 85.8%), node-negative (n = 49, 49.5%), grade 3 (n = 66, 66.7%), and presented a high Ki67 (n = 74, 74.7%). The distribution of tumor stage according to TNM staging version VI (AJCC) was: I (n = 32, 32.3%), II (n = 37, 37.4%), III (n = 30, 30.3%).

All patients received neo/adjuvant chemotherapy, namely 33 patients underwent neoadjuvant treatment, and we evaluated the analysis of the biomolecular determinants by immunohistochemistry on surgical samples. The most frequently used chemotherapy regimens were anthracycline and taxanes based-therapies.

At a median follow-up of 62.0 months (range 3.0-118.0), 95 patients were evaluable for RFS and OS and the 47.5% of cases presented a loco-regional and/or distant recurrence. The most frequent sites of metastases were the following: liver (19%), lung (17%), skin/soft tissue (9%) and distant lymph nodes (6%). Moreover, 34% of patients presented multiple metastatic sites.

AR, CDH1 expressions were found in 17.1% and 50.5% of the cases. The 74.7% of patients showed high Ki67 levels. In 53/99 patients the CK5/6 expression was evaluated and in 54.7% of cases this expression resulted positive [Table 2]. The absence of AR expression was significantly associated with highly undifferentiated tumors: only 26.6% of AR-positive cases were grade 3 compared with 68.2% of grade 3 in AR-negative cases (*p*=0.01). Univariate analyses showed that lack of expression of CDH1, tumor size and nodal status were significantly associated with worse RFS and OS (*p*< 0.05). AR expression and low Ki67 showed a trend towards better RFS and OS [Table 3]. IHC expression of CK5/6 was analyzed in a small sample of patients (53/99) and the absence of expression was associated in the univariate and multivariate analyses with poor RFS (*p*=0.02 and *p*=0.002, respectively) [Table 3] and OS (*p*=0.05 e *p*=0.02, respectively) [Table 4].

**Table 2:** Molecular determinants in our cohort of patients

**CDH1**	
Positive	50 (50.5%)
Negative	49 (49.5%)
**AR**	
Positive	17 (17.1%)
Negative	82 (82.9%)
**Ki67 index ≥20%**	74 (74.7%)
**CK5/6**	
Positive	29 (54.7%)
Negative	24 (45.3%)

**Table 3:** Univariate analyses for RFS and OS using all variables

	RFS		OS	
	HR	p-value	HR	p-value
AR-positive	0.34	0.07	0.67	0.32
CK5/6-positive	0.39	0.02	0.48	0.05
Tsize*				
1	1	-	1	-
2	1.75	0.10	1.41	0.24
3	4.68	<0.01	2.48	0.02
Node-positive	2.00	0.03	1.84	0.04
Grade				
1-2	1.00	-	1.00	-
3	2.03	0.06	1.96	0.03
CDH1-positive	0.43	0.01	0.49	0.01
Ki67-low	0.46	0.08	0.56	0.09
Age (cont. variable)	1.01	0.39	1.03	0.01

*Legend:* *Tsize 1: tumor size ≤2 cm; Tsize2: tumor size >2 cm, but ≤5 cm; Tsize3: tumor size ≥5 cm.

**Table 4:** Multivariable analyses for RFS and OS using selected variables

	RFS		OS	
	HR	p-value	HR	p-value
AR-positive	-	-	-	-
CK5/6-positive	0.23	<0.01	0.39	0.02
Tsize				
1	1	-	1	-
2	1.91	0.17	1.11	0.81
3	4.76	0.02	0.66	0.56
Node-positive	0.88	0.78	1.22	0.66
Grade				
1-2	1.00	-	1.00	-
3	3.09	0.02	3.30	0.01
CDH1-positive	-	-	-	-
Ki67-low	-	-	-	-
Age (cont. variable)	-	-	-	-

Multivariate analysis showed an independent association between CDH1 expression and better RFS and OS (*p*< 0.05) beyond tumor size, nodal status and grade [Table 5]. Multivariate analyses for RFS and OS for all variables analyzed are shown in Table 6.

**Table 5:** Multivariable analyses for RFS and OS using selected variables

	RFS		OS	
	HR	p-value	HR	p-value
AR-positive	-	-	-	-
CK5/6-positive	-	-	-	-
Tsize				
1	1	-	1	-
2	2.01	0.06	1.41	0.27
3	6.10	<0.01	2.52	0.04
Node-positive	1.35	0.38	1.26	0.45
Grade				
1-2	1.00	-	1.00	-
3	1.59	0.38	1.43	0.45
CDH1-positive	0.48	0.04	0.47	0.01
Ki67-low	-	-	-	-
Age (cont. variable)	-	-	-	-

**Table 6:** Multivariable analyses for RFS and OS using all variables

	RFS		OS	
	HR	p-value	HR	p-value
AR-positive	0.17	0.06	0.58	0.40
CK5/6-positive	0.10	<0.01	0.35	0.07
Tsize				
1	1	-	1	-
2	1.05	0.92	0.87	0.78
3	4.83	0.04	0.88	0.85
Node-positive	1.08	0.88	1.07	0.90
Grade				
1-2	1.00	-	1.00	-
3	2.48	0.10	3.03	0.03
CDH1-positive	0.63	0.54	0.35	0.04
Ki67-low	0.62	0.49	0.92	0.90
Age (cont. variable)	0.97	0.10	1.02	0.32

**Figure 1:**
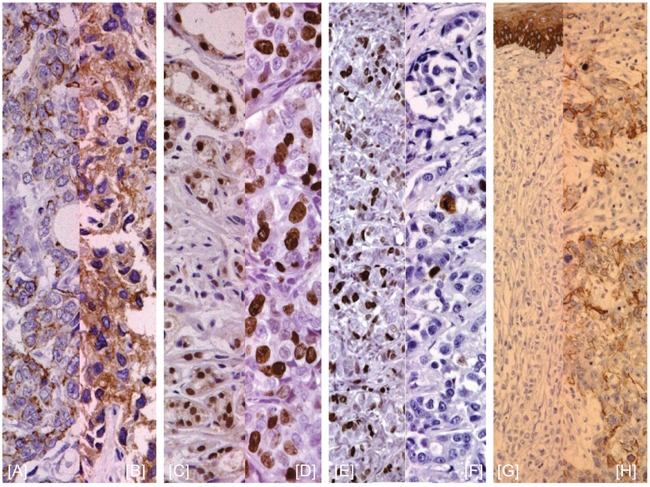
E-Cadherin, Ki67, AR, and CK5/6 expression by IHC **(A**, **B)** E-cadherin negative/positive staining; **(C**, **D)** Ki-67 level < or ≥ 20%; **(E**, **F)** negative/positive AR staining; **(G**, **H)** negative/positive CK5/6 staininig.

The Kaplan-Meier curves showed that patients with AR and CDH1 negative expression and high Ki-67 levels have a significant correlation with poor outcome [Figure 1].

## DISCUSSION

Here we present the outcome of further additional cases updating the previous study [25] in which we evaluated the prognostic role of different molecular determinants, including Androgen receptor (AR), E-Cadherin (CDH1), Ki-67, and CK5/6.

Androgens play a role in breast cancer and several studies showed that the androgen receptor expression in ER-negative tumors can induce proliferative effect and promote tumorigenesis [26]. Although AR is expressed approximately in 10-15% of TNBCs [27], it represents an emerging and a new prognostic marker. The prognostic role of AR expression has reported controversial results in TNBC, likely owing to differences in the antibodies used, scoring systems and cut-off values used for the estimation of AR positivity [28], albeit recent meta-analyses support a possible positive prognostic role for AR-expression in ER-negative BCs [29, 30]. In our analysis, we demonstrated that AR was expressed in 17.1% of the cases and that absence of AR expression was significantly associated with aggressive behavioral tumor and a shorter RFS and OS. Similar findings have been previously reported in our preliminary study in a small sample (45 TNBC patients), suggesting that AR expression is an important prognostic marker in TNBC, since lack of AR expression was associated with a worse outcome in the multivariate analysis [25]. These data are in line with the results of Asano et al. and Jiang et al. that suggested a favorable prognostic role for AR expression in TNBC [31, 32], as further confirmed by a recent meta-analysis [33]. Moreover, another small retrospective study reported that AR expression is associated with chemo-resistance to NAT, albeit these data need further confirmation in large prospective studies [34].

Preclinical data demonstrated that LAR cell lines (SUM185PE, CAL-148, MDA-MB-453, and MFM-223) are sensitive to AR antagonists such as bicalutamide [21], suggesting that LAR TNBC subtypes may benefit from AR-targeted therapies. Indeed, the AR signaling pathway represents an emerging oncogenic driver in AR-expressing TNBCs that may extend the use of AR inhibitors among patients with TN tumors, especially those not responding to chemotherapy [28].

These data provided the rationale for pharmacological targeting of AR signaling pathway in AR-positive TNBCs. Recently, a phase II study with bicalutamide in AR-positive TNBCs was published, reporting an intriguing 6-months Clinical Benefit Rate (CBR) of 19% and a median PFS of 12 weeks, with a relatively favorable safety profile [35]. The role of newer anti-androgen agents, including Abiraterone and Enzalutamide has been evaluated in preclinical models and in phase 2 trials.

The activity of Enzalutamide, a second-generation AR antagonist with proven efficacy in the treatment of castration resistant prostate cancer (CRPC), demonstrated intriguing preclinical activity in AR-positive TNBC models [36–38] and was evaluated in a phase 2 trial in a TNBC population. This study reported an interesting 16-weeks CBR of 35% and median PFS of 14.7 weeks. Further analysis of this study showed a greater benefit in terms of CBR at 16 weeks of 39% and in terms of median PFS of 16 weeks in patients with an androgen-driven gene signature (AR-predict) [39]. Another potent AR inhibitor, Abiraterone acetate, was recently evaluated in association with prednisone in a phase 2 trial reporting a CBR at 6-months of 20.0% and median PFS of 2.8 months [40]. TAK-700 (Orteronel), an oral, non-steroidal androgen synthesis inhibitor that selectively inhibits the 17,20 lyase enzyme is under evaluation in AR+ TNBC [41]. Another possible pharmacological strategy is targeting alternative pathways implicated in ligand-independent AR activation either as single agents or combined with anti-androgens, including inhibitors of PI3K/mTOR, ERK1/2, EGFR and PDGFR-β, as well as LHRH agonists. Results from preclinical studies revealed the presence of a ligand-independent activation of AR signaling through JAK/STAT3, MAPK, NOTCH and PI3K/mTOR/AKT signaling pathways [42, 43]. Moreover, it has been reported that the majority of AR-positive TNBCs carry PIK3CA mutations and/or loss of PTEN, two well-known key components of the PI3K/mTOR pathway, resulting in a constitutive activation of this signaling pathway [44, 45]. These data provided the rationale for evaluation of Enzalutamide in combination with a PI3K inhibitor, Taselisib, in an ongoing, phase Ib/II clinical trial in selected AR-positive TNBCs [NCT02457910].

Ki67 is a nuclear protein, commonly used as proliferative marker, since its expression varies throughout the cell cycle, with peak expression during mitosis. The role of this protein as predictive and prognostic marker has been extensively studied in breast cancer, although there is no standard cut-off definition to date [46]. The recent St Gallen Consensus 2015 has selected optimal cut-off values of Ki67 according to median of Ki67 by local laboratory, defining values ≥30% clearly high and values of ≤10% low [47].

Several studies have reported a positive correlation between increased rates of pathological complete response (pCR) after neoadjuvant chemotherapy (NAT) and high Ki67 levels, suggesting a possible role as predictive marker of response to NAT [48]. Moreover, various studies showed that higher Ki67 levels correlated with worse prognostic factors [49, 50]. Abdlazem et al. have reported a negative correlation of AR expression and low Ki67, showing that tumors with lower Ki67 expression were AR positive. This is probably related to the anti-proliferative effect of AR. Indeed, these tumors are associated with a better prognosis [51]. Keam et al. have evaluated the possible prognostic and predictive role of Ki67 identifying two subgroups of TNBC according to Ki67 levels, reporting that high Ki67 values ≥10% were associated with a higher pCR rate [52].

Our study confirms that lower levels of Ki67 are associated with tumors with less aggressive behavior. In addition, we reported a trend towards better RFS and OS with concomitant AR expression and low Ki67.

E-cadherin is a transmembrane glycoprotein synthesized by the CDH1 gene located in chromosome 16q22.1 playing a significant role in cell proliferation regulation, invasion and metastasis suppression [53, 54]. Loss of E-cadherin has been related to larger tumor size, higher tumor grade, and higher incidence of metastasis in BC [55, 56]. Downregulation of E-cadherin expression represents an epithelial-to-mesenchymal transition hallmark [57] and is associated with chemoresistance in TNBC. Few studies have evaluated, to date, the prognostic role of E-cadherin expression in TNBCs. Kashiwagi et al. and Shen et al. showed that negative CDH1 expression is associated with worse prognosis in TNBC patients [58, 59]. Tang et al. correlated AR and E-cadherin co-expression with different clinic-pathological variables in 127 TN patients, demonstrating that highly undifferentiated tumors and menopausal status were associated with an AR-negative status (p = 0.017) and that positive lymph node status was associated with lack of E-cadherin expression (p = 0.016). Moreover, the absence of AR and loss of E-cadherin expressions were significantly associated with worse DFS (p = 0.047 and p = 0.016, respectively) and OS (p = 0.038 and p = 0.012 respectively) [60].

Unfavorable prognosis significance of the loss of E-cadherin expression has been demonstrated in our study. In our preliminary report, univariate analysis demonstrated that the CDH1, AR and Ki67 co-expression were significantly associated with a better outcome [25]. In this final analysis, conducted on a larger sample of TNBC patients, the CDH1 expression was found in the 50.5% of cases and the statistical analysis shows a significant correlation between lack of E-cadherin expression and worse outcome. In particular, the multivariate analysis demonstrated that the E-cadherin expression is an independent prognostic variable of longer RFS and OS (p< 0.05) beyond tumor size, nodal status, and tumor grade.

Basal CKs are intermediate filaments present in the myoepithelial and basal epithelial cells of the mammary gland. Basal CKs have been described in many cancer types, including breast tumors.

However, the interest in basal CKs rapidly increased after the identification by Perou et al. of a ‘‘*basal-like’’* subgroup of breast carcinomas characterized by a more aggressive phenotype and by expression of genes normally active in the basal/myoepithelial cells [61–63].

In a small cohort of patients (53/99), we also evaluated the role of basal cytokeratins CK5/6 expression in TNBCs. CK5/6 were expressed in 54.7% of the tumors and were associated with worse RFS and OS in both univariate and multivariate analyses. The negative prognostic role of CK5/6 suggests that the population of the study with CK5/6 expression might belong to the basal-like subtype. Recently, Maeda et al. reported that the absence of expression of CK5/6, AR and the presence of p53 is associated with a poor prognosis after adjuvant chemotherapy in 52 TNBCs [64].

Given their relative easy estimation and based on the results presented here, we suggest the implementation of the use of these molecular determinants in clinical practice to allow the identification of specific subgroup of patients with more aggressive behavior.

## MATERIALS AND METHODS

A total of 99 TNBC patients who underwent surgical resection at two Italian Hospital Centers between January 2000 and December 2010 were included in this retrospective, observational study.

This study was conducted on archived tumor section and informed consent was obtained from donors or the next of kin.

The eligibility criteria were: women aged ≥18 years; histological diagnosis of breast cancer (stage I–IV according to TNM [tumor, node, metastasis] American Joint Committee on Cancer [AJCC] version VI); availability of the following local staging and biological parameters: pT, pN, grade (G); all patients underwent surgical resection such as a mastectomy or conservative surgery with axillary lymph node dissection. The patients were considered triple negative if the ER and PgR cell staining of both were 0% by IHC, and HER2 staining of 0 by IHC or 1+ and 2+ score with no gene amplification by fluorescence *in situ* hybridization (FISH), according to the last version of the American Society of Clinical Oncology (ASCO)/College of American Pathologists (CAP) guidelines [65, 66].

Patients with either expression of ER/PgR or overexpression/amplification of HER2 or metastatic disease were not included in this analysis.

We assessed the expression of AR, CDH1, Ki67 and CK5/6 in primary breast cancer by immunohistochemistry (IHC).

Sections were cut from formalin-fixed, paraffin-embedded specimens of the primary tumors for staining. Tissue sections were then incubated with each primary monoclonal antibody against AR (clone AR441; Dako; diluition 1:100), E-Cadherin (clone NCH-38, Dako; diluition 1:200), Ki67 (clone MIB-1, Dako; diluition 1:100), and CK5/6 (D5/16B4, Dako; diluition 1:100).

**Figure 2:**
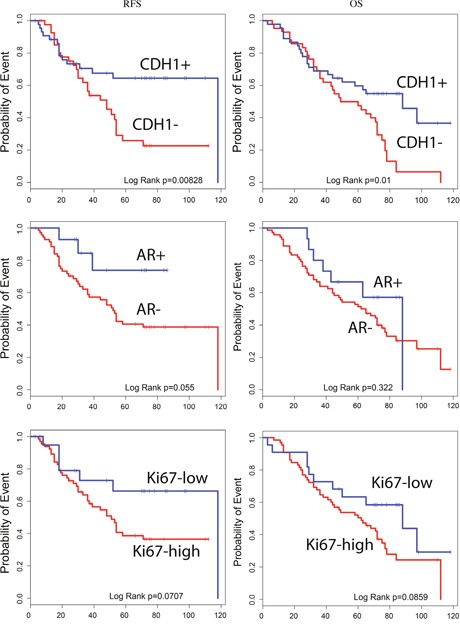
Estimated RFS and OS curves for CDH1, AR and Ki67 expression

Sections were considered AR positive when ≥10% of tumor cell nuclei stained positive. E-cadherin expression was semi-quantitatively analyzed according to the percentage of cells showing membrane positivity: 0 (0 to 10%); 1+ (10 to 30%); 2+ (30 to 70%); 3+ (>70%). E-cadherin expression was considered positive if the score was ≥ 2, and negative when score was ≥1. [Figure 2]

We considered a high Ki67 index > 20%. CK5/6 expression was considered positive if the score was ≥1.

### Statistical analysis

The correlation among different clinical-pathological parameters with biomarkers expression was evaluated by Chi squared test. In addition, the association between the various clinical-pathological variables (i.e. androgen receptor expression, CDH1 status, Ki 67 expression, histological type, histological grade, lymph nodal status) and clinical outcomes were estimated through both univariate and multivariate analyses. Univariate and multivariate analyses were conducted using Cox proportional hazards regression and the results were considered statistically significant with a p value <0.05.

The relapse-free survival (RFS) was defined as the interval, in months, from the date of the diagnosis to the first local recurrence or distant metastasis. The overall survival (OS) was the time, in months, from the date of the diagnosis to the time of breast cancer-related death or death from any cause.

The survival curves were estimated using the Kaplan-Meier method and differences between groups were evaluated using the log-rank test.

All analyses were performed using R package 2.15.1 (http://www.r-project.org).

## CONCLUSIONS

TNBC is an aggressive BC subtype with a poor prognosis. Indeed, despite the extensive efforts conducted to better comprehend the molecular biology of these tumors, there are no effective targeted therapies for this subtype to date and limited therapeutic options are available for such patients.

Given the high heterogeneous nature of TNBC, there is an urgent clinical need to identify and develop valid biomarkers that reflect the different behavior of this subtype. Moreover, increasing evidence shows that the presence or not of a specific molecular phenotype related to the expression a variable number of biomarkers confers the tumor a different degree of aggressiveness.

Albeit limited by its retrospective nature, our study supports the use of IHC expression of AR, CDH1, Ki67 and CK5/5 as prognostic markers in TNBCs and suggests a link between the expression of these molecular determinants and prognosis and may help to stratify TNBC patients in different prognostic classes. Our data provide clinical evidence that TNBCs could be further divided into two classes of prognosis subtypes (positive and negative) according to AR, E-cadherin, Ki67 and CK5/6 expressions. Thus, clinical implementation of the IHC expression of these molecular determinants may represent an important tool for the stratification of TNBCs in different prognostic groups with variable outcome.

Our findings suggest that AR, E-cadherin, Ki67 and CK5/6 expression may be novel and promising biomarkers in TNBC and could be useful prognostic markers for classifying subgroups of TNBCs.

Further development of this study will provide the extension of the analysis of basal cytokeratins CK5/6 to the entire cohort of TN patients and the integration of clinico-pathological and immunohistochemical data with the analysis of gene expression profiles trought PAM50 in order to confirm the clinical utility of these pathological factors in the prognostic stratification of TNBC patients.

## Abbreviations

AJCC: American Joint Committee on Cancer
AR: Androgen Receptor
ASCO/CAP: American Society of Clinical Oncology/College of American Pathologists
BC: Breast Cancer
BL: basal-like
BLIA: basal-like immune-activated
BLIS: basal-like immunosuppressed
BRCA: Breast Related Cancer Antigens
CBR: Clinical Benefit Rate
CDH1: Cadherin-1 or epithelial cadherin (E-cadherin) gene
CK5/6: Cytokeratin 5/6
CRPC: Castration-Resistant Prostate Cancer
DFS: Disease-free Survival
DMFS: Distant Metastasis-free Survival
EMT: Epithelial-Mesenchymal-Transition
ER: Endocrine Receptor
ERK: Extracellular Signal–regulated Kinase
FDA: Food Drug Administration
FISH: Fluorescent *in situ* hybridization
GEP: Gene Expression Profile
HR: Hazard Ratio
IGF-1: Insulin-like Growth Factor 1
IHC: Immunoistochemistry
IM: Immunomodulatory
JAK: Janus Kinase
LAR: Luminal Androgen Receptor
LHRH: Luteinizing Hormone–Releasing Hormone
MES: Mesenchymal
mTOR: mammalian target of rapamycin
MUC1: Mucin 1, cell surface associated
NAT: Neo-adjuvant chemotherapy
OS: Overall Survival
pCR: pathological complete response
PDGFR-β: Platelet-derived growth factor receptor-β
PI3K: Phosphatidylinositol-4,5-bisphosphate 3-kinase
RFS: Relapse-free survival
STAT: Signal Transducer and Activator of Transcription
TGCA: The Cancer Genome Atlas
TNBC: Triple Negative Breast Cancer
TNM: Tumor, Node, Metastasis
VTCN1: V-Set Domain Containing T Cell Activation Inhibitor 1
