# Correction: The prognostic significance of combined androgen receptor, E-Cadherin, Ki67 and CK5/6 expression in patients with triple negative breast cancer

**DOI:** 10.18632/oncotarget.26650

**Published:** 2019-01-25

**Authors:** Barbara Adamo, Giuseppina Rosaria Rita Ricciardi, Antonio Ieni, Tindara Franchina, Carmine Fazzari, Maria Vita Sanò, Giuseppe Angelico, Michele Caruso, Giovanni Tuccari, Vincenzo Adamo

**Affiliations:** ^1^ Department of Medical Oncology, Hospital Clínic of Barcelona, Barcelona, Spain; ^2^ Medical Oncology Unit A.O. Papardo & Department of Human Pathology University of Messina, Messina, Italy; ^3^ Department of Human Pathology of Adult and Evolutive Age “Gaetano Barresi”, Section of Pathology, University of Messina, AOU Policlinico “G. Martino” Messina, Italy; ^4^ Pathology Unit, Humanitas Center of Oncology, Catania, Italy; ^5^ Medical Oncology, Humanitas Catania Oncology Center, Catania, Italy; ^6^ G. F. Ingrassia Department, Section of Anatomic Pathology, University Hospital “Policlinico-Vittorio Emanuele”, Catania, Italy

**This article has been corrected:** The correct author name and author contributed information is given below:

Barbara Adamo^1,*^, Giuseppina Rosaria Rita Ricciardi^2,*^, Antonio Ieni^3^, Tindara Franchina^2^, Carmine Fazzari^4^, Maria Vita Sanò^5^, Giuseppe Angelico^6^, Michele Caruso^5^, Giovanni Tuccari^3^ and Vincenzo Adamo^2^

^*^ These authors have contributed equally to this work

Original article: Oncotarget. 2017; 8:76974-76986. https://doi.org/10.18632/oncotarget.20293

